# Unique Presentation of an Arteriovenous Malformation (AVM) in the Hand: A Case Report

**DOI:** 10.7759/cureus.114724

**Published:** 2026-08-18

**Authors:** Hasan A Al-Humairi, Mohammed A Alsayed, Mohammed R Sajwani, Yasir A Al-Humairi, Hamed Badawi

**Affiliations:** 1 Medicine and Surgery, University of Sharjah, Sharjah, ARE; 2 Orthopedics and Trauma, Rashid Hospital, Dubai, ARE; 3 Hand Surgery, Rashid Hospital, Dubai, ARE

**Keywords:** arterio venous malformations, embolization, hand avm, ray amputation, vascular steal

## Abstract

Arteriovenous malformations (AVMs) of the hand are challenging high-flow vascular anomalies. While often stable for years, they can progress into higher Schobinger stages, where ulceration, hemorrhage, distal ischemia, and, in advanced cases, congestive heart failure are characteristic. Incomplete prior interventions often exacerbate these outcomes.

A 39-year-old male with a 12-year history of a stable hand AVM presented with sudden expansion, spontaneous hemorrhage, and gangrenous skin changes. Clinical evaluation revealed hematoma and vascular steal syndrome. Imaging (MRI and DSA) confirmed a large (8x7x6 cm) extratruncular high-flow AVM involving the volar and ulnar aspects of the hand. The patient was managed with intravenous antibiotics and a staged multidisciplinary approach including selective arterial embolization with mechanical coils and liquid embolic agents, followed by radical surgical excision 48 hours later.

Advanced hand AVMs require a "total obliteration" strategy. This case highlights the success of combining endovascular modulation with surgical resection to achieve limb salvage in a stage III lesion. Lifelong surveillance is mandatory due to the high risk of recurrence from dormant microscopic vascular nests.

## Introduction

Arteriovenous malformations (AVMs) are high-flow vascular anomalies characterized by direct communication between arteries and veins without an intervening capillary bed. AVMs are congenital in nature, but they often show no symptoms for years [1,2]. These malformations are either found incidentally or found only after becoming clinically apparent in approximately 20% of cases [1]. These lesions are dynamic and can be exacerbated by external triggers such as trauma, hormonal shifts during puberty or pregnancy, or previous iatrogenic triggers such as incomplete interventions [3]. AVMs affecting the extremities and particularly the hands carry significant risks of progressive deformation, chronic pain, and severe functional impairment [1].

The clinical severity and progression of AVMs are traditionally categorized using the Schobinger staging system [1]. Symptoms typically progress from a simple cutaneous blush (stage I) to the development of thrills, bruits, and expansion (stage II). When complications such as ulceration, intractable pain, or life-threatening hemorrhage occur, the lesion is classified as stage III. Further progression can lead to stage IV, characterized by high-output congestive heart failure [2]. In the hand, these lesions may also cause "arterial steal" and venous hypertension, potentially leading to distal ischemia and gangrenous changes in the digits [2].

Management of AVMs in the hand is notoriously difficult due to the dense concentration of vital neurovascular structures and the high risk of recurrence. There is no single consensus on the best therapeutic approach, but many experts advocate for a multidisciplinary strategy. This typically involves preoperative selective angiography and embolization to obliterate the high-flow components and minimize perioperative blood loss, followed by definitive surgical excision [1,3,4].

This report describes the case of a 39-year-old male with a 12-year history of a previously stable hand AVM that acutely progressed to Schobinger stage III, presenting with spontaneous hemorrhage. This report highlights the method of approaching such vascular anomalies and the success of a staged approach combining selective embolization and surgical resection.

## Case presentation

A 39-year-old male presented to the emergency department with complaints of left hand pain and recurrent bleeding from a known vascular lesion. The patient reported that the lesion had been present for approximately 12 years and had remained stable during that time. However, one week prior to presentation, he experienced a sudden increase in size, worsening pain, and discoloration of the lesion. On the day of admission, he developed spontaneous bleeding, prompting emergency evaluation.

The patient had a history of prior vascular intervention for the same lesion approximately 10 years earlier, including incomplete embolization. He had no significant past medical history and was not on regular medications.

On examination, a vascular mass was noted on the left hand, associated with tenderness and swelling. The overlying skin showed discoloration. Distal neurovascular examination revealed intact sensation, palpable pulses, and normal capillary refill. Initial bleeding was controlled with compression.

**Figure 1:**
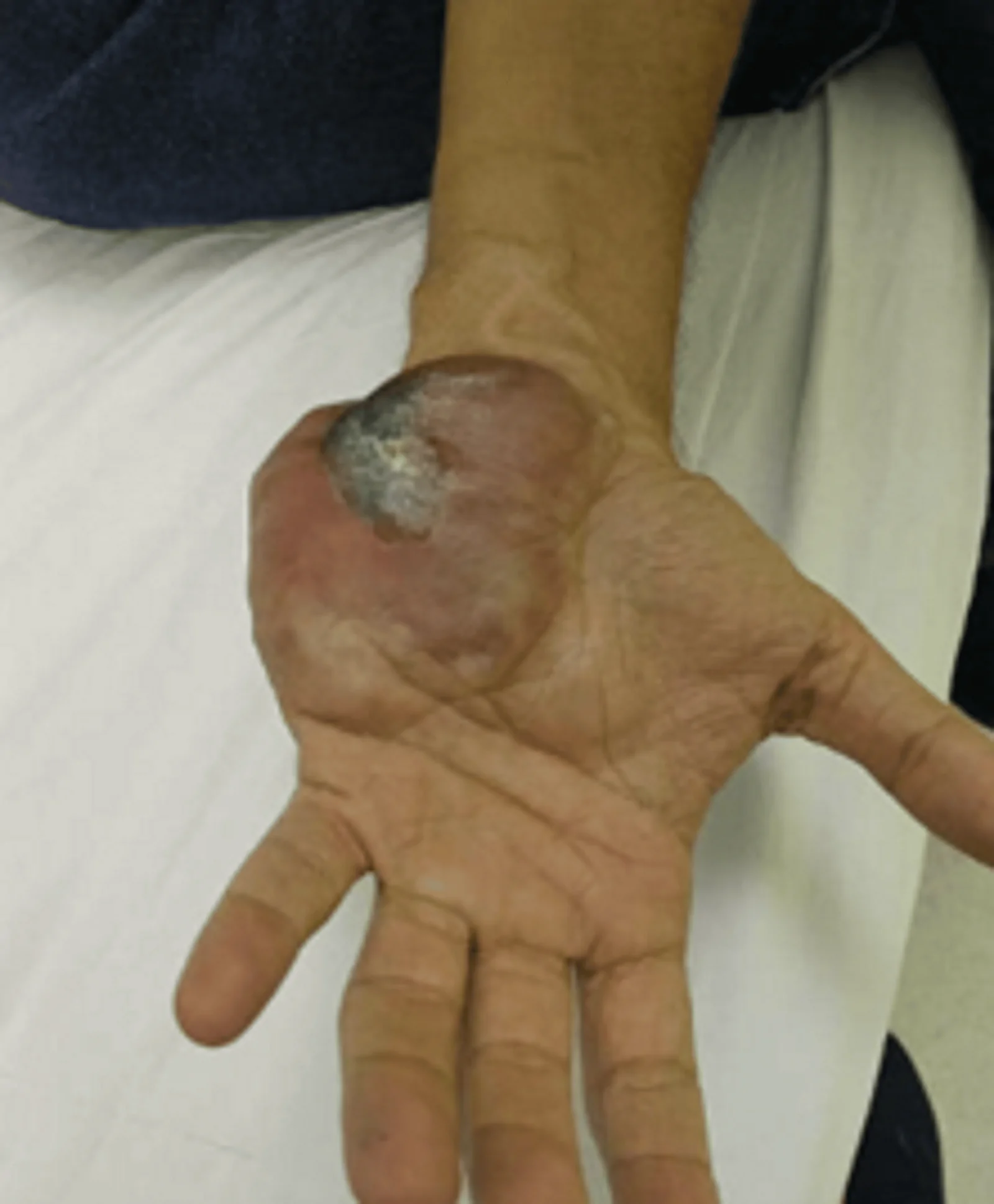
Left hand on presentation on day one

During subsequent visits, the patient developed worsening swelling, erythema, and localized warmth. Areas of skin discoloration progressed to gangrenous changes (Figure 1).

Laboratory investigations demonstrated mild anemia and mildly elevated inflammatory markers, including C-reactive protein. Other labs done, including liver function tests, renal function tests, and coagulation profile, were within normal limits (Table 1).

**Table 1:** Complete blood count and C-reactive protein Low hemoglobin, hematocrit, and RBCs with other parameters within range indicate a normocytic, normochromic anemia. CRP was mildly elevated, indicating an active inflammatory process. RBC - red blood cells; WBC - white blood cells; MCV - mean corpuscular volume; MCH - mean corpuscular hemoglobin; MCHC - mean corpuscular hemoglobin concentration; RDW - red blood cell distribution width; MPV - mean platelet volume; CRP - C-reactive protein

Parameter	Result	Reference Range
RBC	3.65 ×10⁶/µL	4.50-5.50
Hemoglobin	10.3 g/dL	13.0-17.0
Hematocrit	30.9%	40.0-50.0
WBC	8.0 ×10³/µL	3.6-11.0
MCV	82.6 fL	77.0-95.0
MCH	28.2 pg	27.0-32.0
MCHC	34.1 g/dL	31.5-34.5
RDW	15.7%	11.5-14.0
Platelets	209 ×10³/µL	150-410
MPV	9.0 fL	7.4-10.4
Neutrophils	5.00 ×10³/µL	2.00-7.00
Lymphocytes	2.00 ×10³/µL	1.00-3.00
Monocytes	0.60 ×10³/µL	0.20-1.00
Eosinophils	0.20 ×10³/µL	0.00-0.50
Basophils	0.10 ×10³/µL	0.00-0.10
CRP	40.1 mg/L	<5.0 mg/L

**Figure 2:**
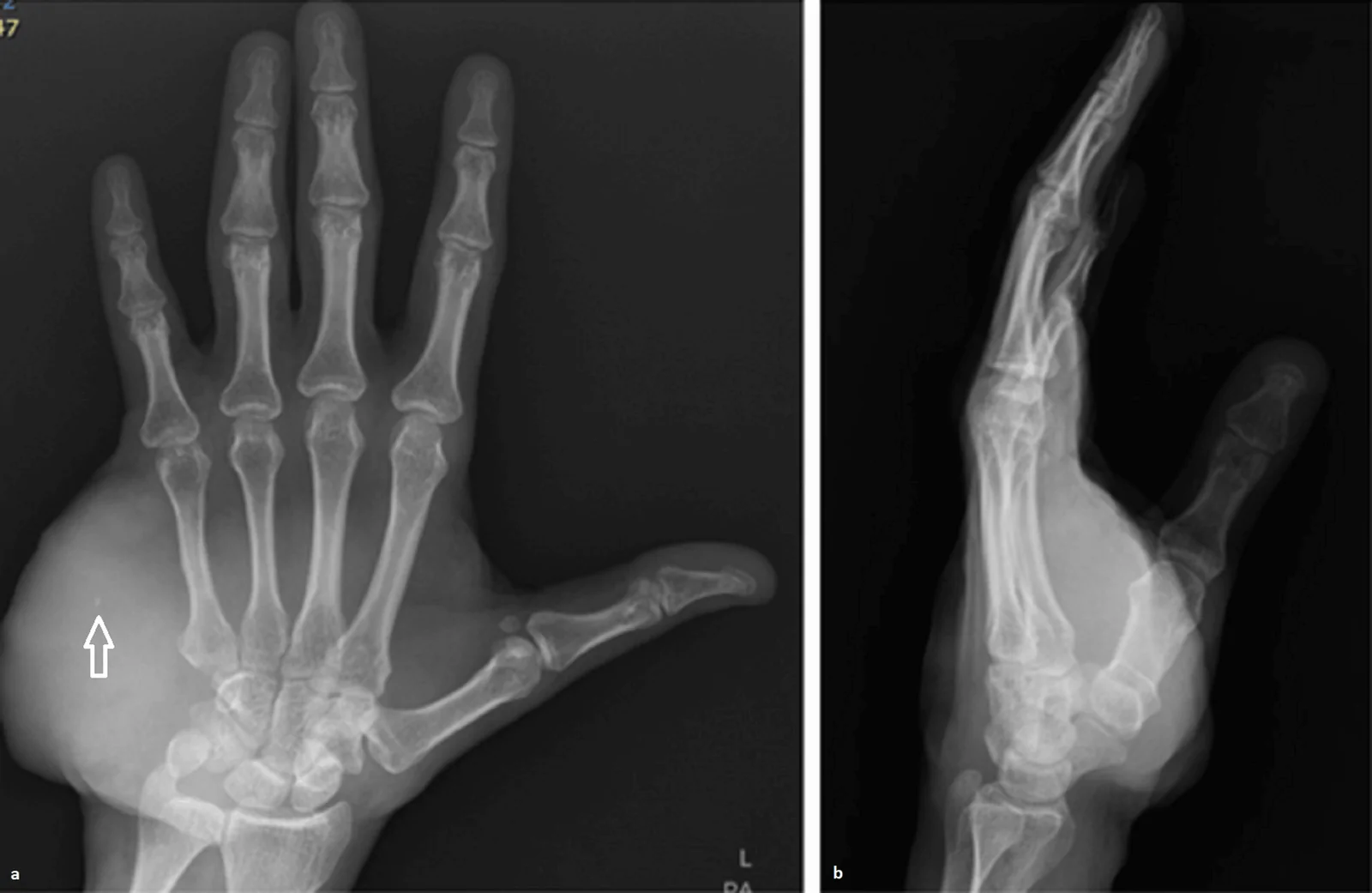
X-ray left hand PA (A) and lateral (B) Lobulated homogeneously dense soft tissue lesion seen along the ulnar aspect of the hand. Small round calcification is noted within the lesion (arrow).

**Figure 3:**
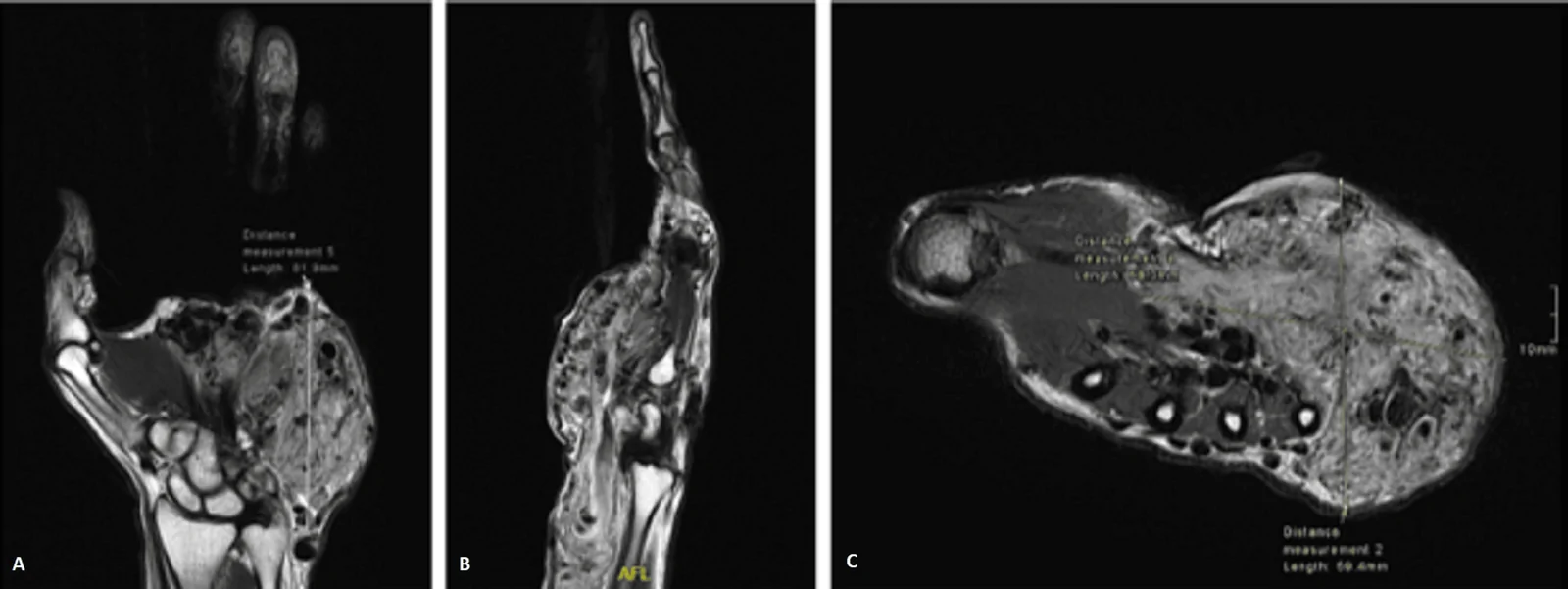
Left hand MRI with coronal (A), sagittal (B) and axial planes (C) Large complex high-flow arteriovenous malformation (AVM) of the left hand involving the volar/ulnar subcutaneous tissues, with arterial feeders from both radial and ulnar arteries and early venous drainage. These vessels extend from the distal forearm into the malformation and continue distally to the level of the proximal digits. The overall malformation and soft tissue complex measures approximately 8 x 7 x 6 cm (CC x TS x AP dimensions).

Ultrasound imaging initially revealed a low-flow arteriovenous malformation. Previous computed tomography had confirmed the diagnosis, and magnetic resonance imaging was performed preoperatively for further evaluation, during which a high-flow arteriovenous malformation was observed (Figures 2, 3).

**Figure 4:**
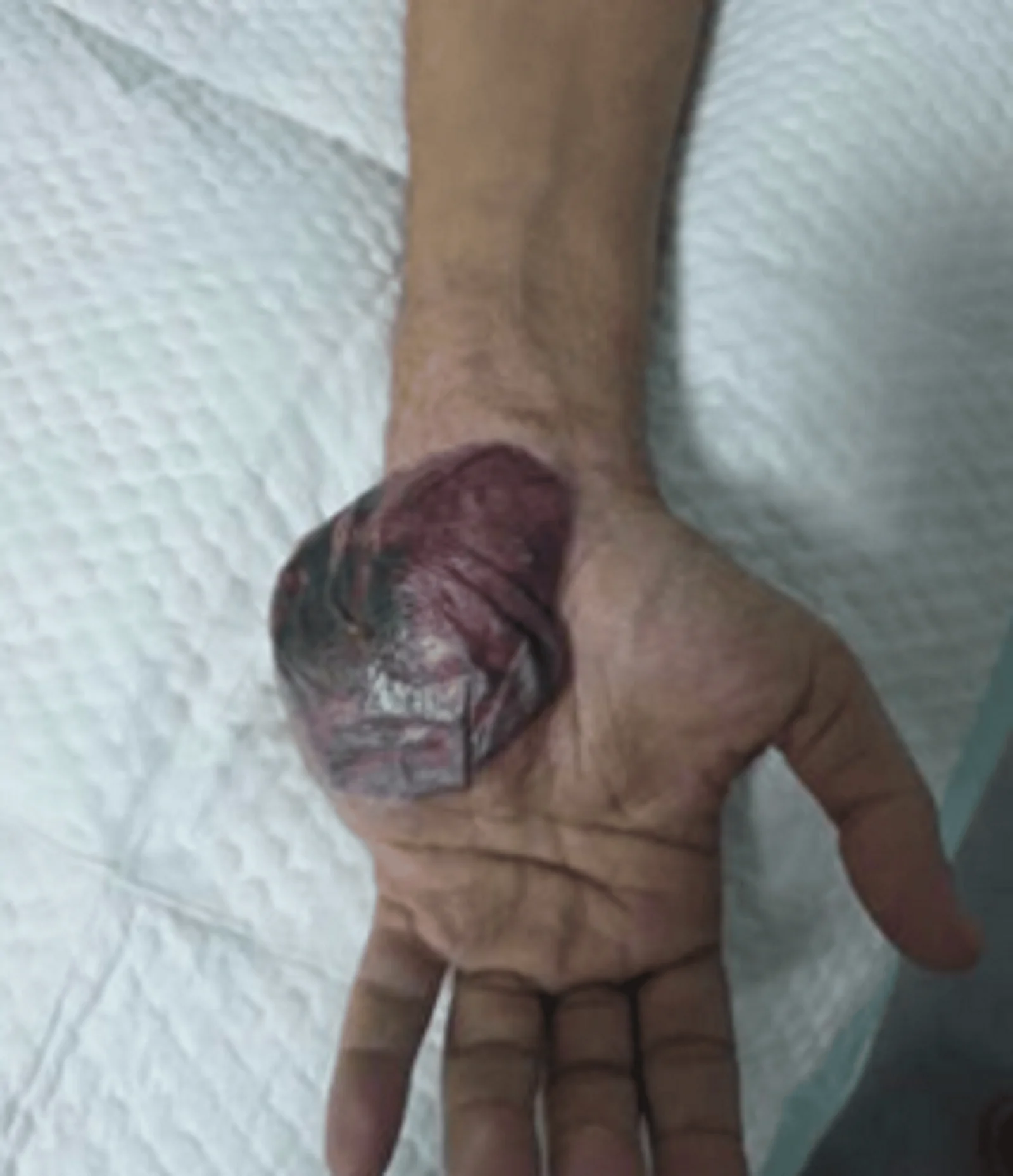
Left hand before intervention on day four

**Figure 5:**
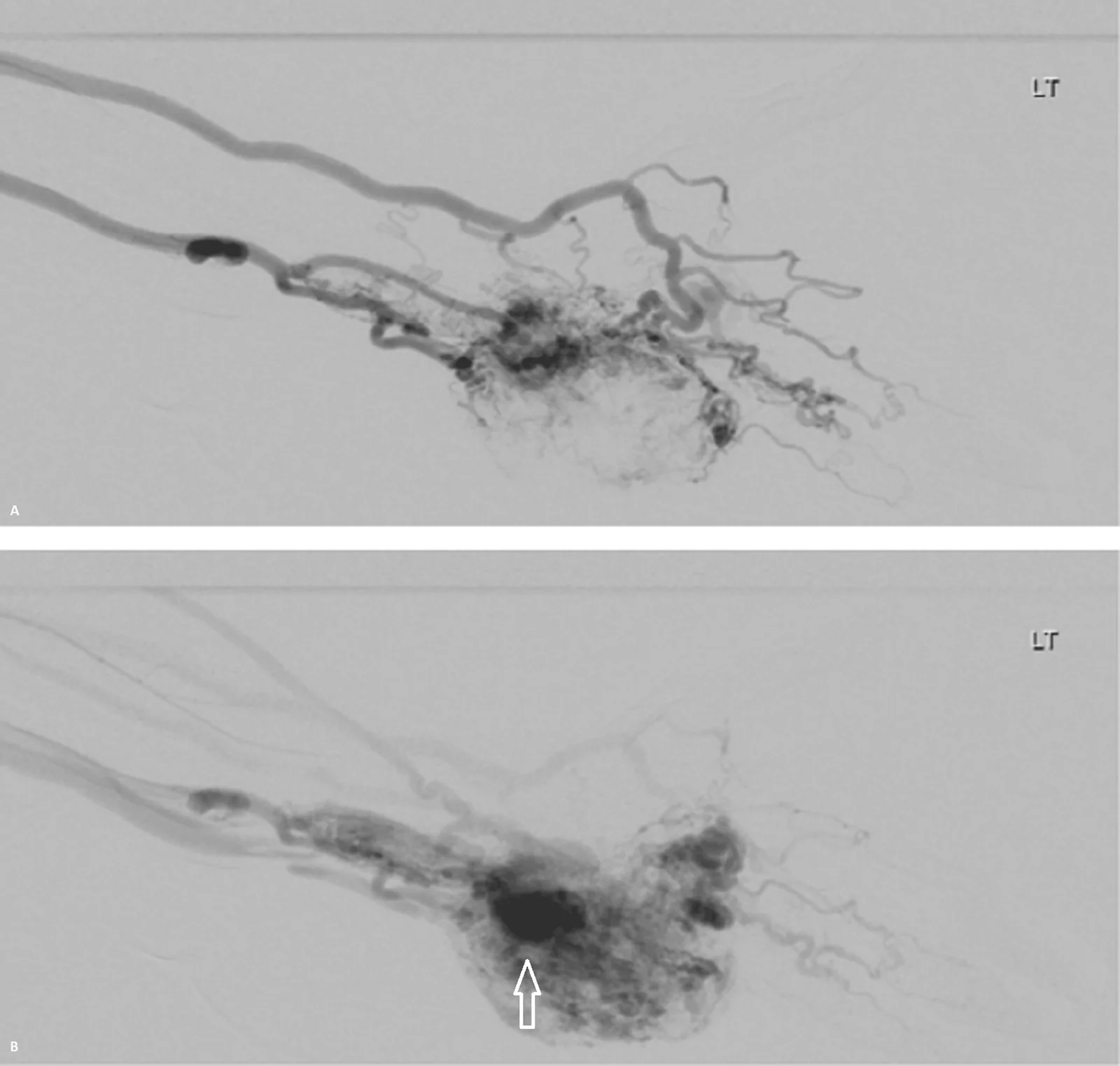
Left upper limb angiogram Early arterial phase demonstrating hypertrophic, tortuous arterial feeders originating from the radial and ulnar arteries supplying the ulnar aspect of the hand (A). Late arterial / early venous phase showing dense opacification of the high-flow vascular nidus (arrow) with rapid, premature venous filling via prominent, dilated draining veins, characteristic of a high-flow arteriovenous malformation with vascular steal (B).

The patient was admitted for further management. Initial treatment included analgesia, compression therapy, and intravenous antibiotics for suspected infection. On day four of admission, the patient underwent selective angiography followed by embolization of the AVM. This resulted in temporary control of bleeding and reduction in vascularity (Figures 4, 5).

**Figure 6:**
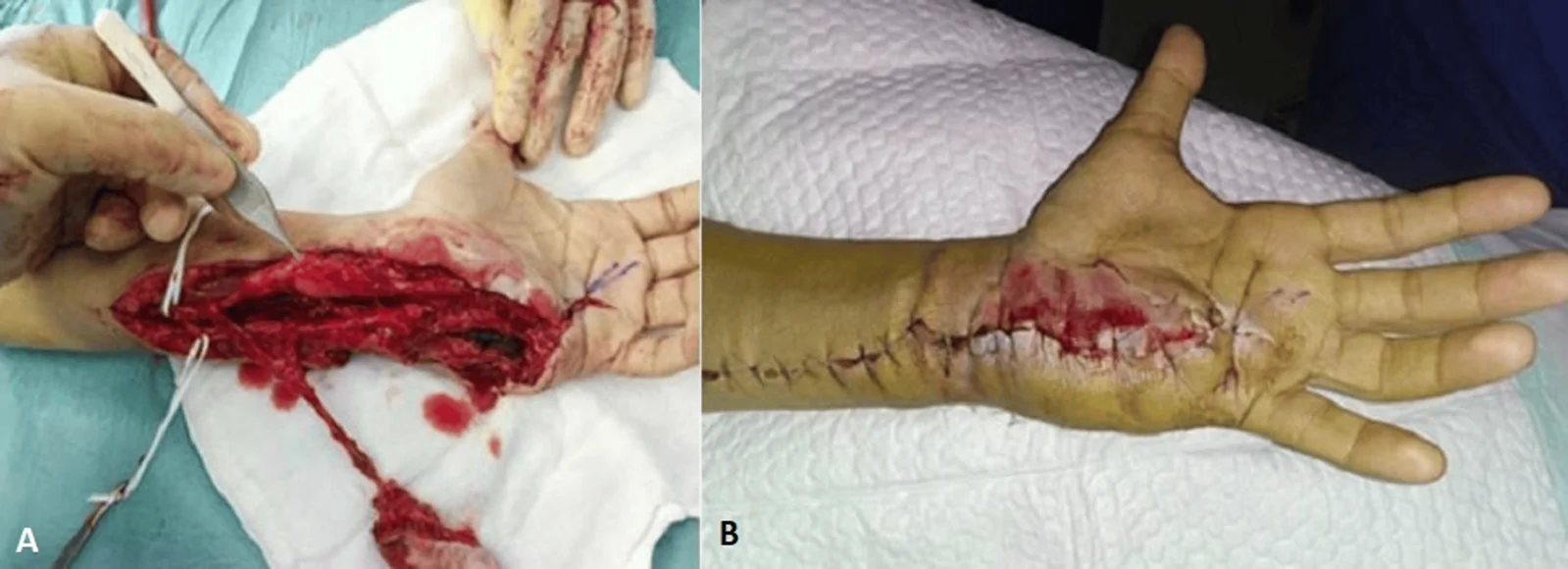
Surgical intervention Surgical exploration and excision of the arteriovenous  malformation (A); surgical wound (B)

Subsequently, on day six, definitive surgical excision of the malformation was performed. Intraoperatively, severe profuse hemorrhage was encountered despite meticulous dissection. To achieve complete hemostasis, surgeons were forced to sacrifice a segment of the ulnar nerve due to intractable bleeding from its intrinsic nutrient vasa nervorum, which persisted despite proximal ulnar artery ligation. Eventually, the bleeding was controlled, and the procedure was completed successfully (Figure 6).

The postoperative period was uneventful, and the patient showed significant clinical improvement, with resolution of pain and no further bleeding episodes. He was discharged on day 13 with planned outpatient follow-up.

## Discussion

Arteriovenous malformations (AVMs) of the hand are enigmatic, therapeutically challenging lesions characterized by high-flow, low-resistance shunts that completely bypass normal capillary networks [5]. Driven by defective embryological vascular morphogenesis and somatic mutations within the RAS/MAPK signaling pathway (e.g., MAP2K1), these lesions trigger a relentless cascade of profound hemodynamic and ischemic alterations [6].

According to the Hamburg classification, extratruncular lesions-such as the massive 8 cm malformation in our 39-year-old patient-exhibit an aggressive tendency to infiltrate surrounding skeletal musculature and neural sheaths, complicating surgical extirpation [7]. Additionally, the International Society for the Study of Vascular Anomalies (ISSVA) classification provides the necessary framework to distinguish these isolated anomalies from syndromic presentations [8].

The clinical trajectory of our patient perfectly illustrates the unrelenting disease progression into Schobinger stage III (destruction). Having harbored the lesion for over a decade, his sudden decompensation into catastrophic hemorrhage, gangrene, and severe neuropathic pain underscores the inherent instability of AVMs. Crucially, this rapid deterioration was likely precipitated by an incomplete embolization attempt a decade prior. Contemporary literature strictly warns that partial obliteration induces acute hypoxia in the residual nidus; this triggers a massive release of angiogenic cytokines (vascular endothelial growth factor, basic fibroblast growth factor) that provoke explosive, chaotic neovascularization and a delayed, aggressive clinical recurrence [9].

The profound tissue destruction seen in advanced AVMs is largely driven by vascular steal syndrome, where blood is diverted or sucked into areas of lower capillary resistance, thus preventing the blood from reaching distal areas with higher capillary resistance. The low-resistance nidus continuously diverts arterial inflow from distal capillary beds, causing chronic hypoperfusion, severe muscular atrophy, and irreversible dry gangrene. Furthermore, the excruciating neuropathic pain experienced by the patient has a dual etiology. Firstly, beyond the regional ischemic monomelic neuropathy caused by vasa nervorum steal, advanced extratruncular AVMs directly infiltrate peripheral nerves. Secondly, dysmorphic vessels physically compress the perineurium and proliferate within the endoneurial space, creating localized edema, rigid compartmental pressure, and continuous aberrant action potentials that render conservative pain management entirely ineffective [10].

As AVMs progress, unrelenting arterial pressure is transmitted directly into thin-walled, "arterialized" draining veins that lack a structural internal elastic lamina [11]. This hemodynamic bombardment causes massive aneurysmal dilation and extreme friability, ultimately leading to spontaneous, life-threatening hemorrhage and consumptive coagulopathy [10]. Furthermore, the massive extravasation of blood into ischemic, ulcerated tissues in our patient resulted in the formation of a hematoma [12]. This limb-threatening complication acts as a potential in vivo culture medium, mandating an immediate shift from elective reconstruction to emergent, broad-spectrum antibiotic therapy and limb-salvaging source control [13].

A sophisticated, multimodal imaging approach is essential for accurate anatomical mapping. While Doppler ultrasound effectively evaluates high-flow shunting, massive compressive hematomas can mechanically dampen flow signals, falsely masking the underlying arterial nature of the lesion [14]. Plain radiography provides baseline data, identifying phleboliths and localized cortical osseous remodeling. Contrast-enhanced MRI remains the gold standard for volumetric assessment, demonstrating classic hypointense "flow voids," mapping the precise relationship to critical neurovascular structures, and quantifying functional complications like retinacular compression. Finally, digital subtraction angiography (DSA) provides the exceptionally high-resolution dynamic visualization of hypertrophic feeders and premature venous drainage that is indispensable for planning targeted embolization [15].

Histopathologically, the AVM nidus is characterized by disorganized, dysmorphic vessels completely devoid of an intervening capillary network. Microscopic hallmarks include severe fibromyxoid intimal thickening and the fragmentation of the internal elastic lamina in feeding arteries, reflecting absolute structural failure under extreme mechanical shear stress [16].

The definitive surgical management of complex extratruncular hand AVMs dictates a highly coordinated multidisciplinary approach. Primary surgical resection without endovascular modulation is exceptionally hazardous due to uncontrollable intraoperative hemorrhage. Preoperative embolization selectively occludes arterial feeders to obliterate the core nidus, drastically reducing shunt volume and creating a hardened, avascular plane for subsequent dissection [17]. In our case, interventionalists successfully deployed mechanical micro-coils to strategically dampen massive arterial inflow velocity, subsequently allowing liquid N-butyl-2 cyanoacrylate (NBCA) glue to polymerize safely within the microvascular nidus without risking catastrophic non-target pulmonary embolization.

Definitive surgery, optimally performed within 24 to 72 hours post-embolization to preempt hypoxia-driven recanalization, requires wide local excision with microscopically negative margins to ensure complete eradication. Advanced hemostatic energy devices are preferentially utilized to secure vessel sealing while minimizing collateral thermal damage to adjacent digital nerves. Management of the massive surgical defect frequently necessitates highly innovative reconstructive techniques, including autologous vein grafts for arterial reconstruction or advanced synthetic nonporous tissue matrices (e.g., acellular dermal matrices) to safely bridge fascial defects [18].

While physical eradication of the nidus immediately resolves the devastating vascular steal and neuropathic pain, the long-term prognosis for Schobinger stage III lesions remains inherently guarded. Due to the diffuse, infiltrative nature of extratruncular AVMs, dormant nests of dysmorphic endothelial cells frequently evade visual detection. The physiological stress of surgical healing can awaken angiogenic pathways, leading to aggressive recurrence rates ranging from 20% to over 40%. Refractory recurrences with intractable pain or bleeding can ultimately require definitive amputation to save the patient's life. Therefore, postoperative management dictates a lifelong, uncompromising surveillance protocol utilizing serial color Doppler ultrasonography and targeted MRI to preemptively detect subclinical recurrent shunting and facilitate minimally invasive early intervention [1]. CT angiography with contrast can also be utilized to identify feeding vessels and guide appropriate interventions.

Our patient presented with an acute, life-threatening spontaneous hemorrhage arising from a chronically expanded hand AVM, compounded by severe intraoperative hemorrhage that mandated ulnar artery ligation and ulnar nerve sacrifice. While digital and limb preservation remain the primary objectives of combined endovascular and surgical therapies, uncontrollable bleeding, extensive tissue destruction, or intractable neuropathic pain in Schobinger stage III lesions may ultimately necessitate aggressive surgical salvage, such as ray amputation [19,20]. In cases involving the medial hand, a partial hand resection involving the fourth and fifth rays serves as an effective life-saving option. By completely removing the infiltrative nidus along with its corresponding metacarpals, ray amputation eradicates the source of catastrophic hemorrhage, resolves vascular steal, and significantly lowers the rate of local recurrence while preserving partial hand biomechanics.

Limitations

This report has several limitations. First, lifelong surveillance is required to confirm permanent eradication, or in case of recurrence, we could perform MRI and CT angiography with contrast to plan our next surgery (whether embolization, re-excision of the AV malformation, or possible amputation). Second, as a single-case report, the results are not broadly generalizable to all hand vascular anomalies. Finally, our assessment of distal tissue perfusion was predominantly clinical; the use of advanced perfusion imaging (such as indocyanine green angiography) could have provided a more objective measure of the resolution of the vascular steal syndrome.

## Conclusions

In conclusion, the management of advanced, high-flow arteriovenous malformations of the hand requires a highly coordinated, multidisciplinary approach. As demonstrated in this case, partial interventions are strictly contraindicated, as they often precipitate rapid clinical deterioration, severe vascular steal, and life-threatening complications. The definitive treatment paradigm relies on targeted preoperative endovascular embolization to minimize surgical morbidity, followed swiftly by radical, microscopically precise surgical extirpation. Due to the inherently aggressive, infiltrative nature of these extratruncular lesions and notoriously high long-term recurrence rates, successful limb salvage does not mark the end of treatment, but rather the beginning of rigorous, lifelong clinical and radiological surveillance.
